# Japanese Sound-Symbolic Words for Representing the Hardness of an Object Are Judged Similarly by Japanese and English Speakers

**DOI:** 10.3389/fpsyg.2022.830306

**Published:** 2022-03-15

**Authors:** Li Shan Wong, Jinhwan Kwon, Zane Zheng, Suzy J. Styles, Maki Sakamoto, Ryo Kitada

**Affiliations:** ^1^Division of Psychology, School of Social Sciences, Nanyang Technological University, Singapore, Singapore; ^2^Faculty of Education, Kyoto University of Education, Kyoto, Japan; ^3^Department of Psychology, Lasell University, Newton, MA, United States; ^4^Department of Informatics, Graduate School of Informatics and Engineering, The University of Electro-Communications, Chofu, Japan; ^5^Graduate School of Intercultural Studies, Kobe University, Kobe, Japan

**Keywords:** sound symbolism, hardness, material perception, Japanese, touch

## Abstract

Contrary to the assumption of arbitrariness in modern linguistics, sound symbolism, which is the non-arbitrary relationship between sounds and meanings, exists. Sound symbolism, including the “Bouba–Kiki” effect, implies the universality of such relationships; individuals from different cultural and linguistic backgrounds can similarly relate sound-symbolic words to referents, although the extent of these similarities remains to be fully understood. Here, we examined if subjects from different countries could similarly infer the surface texture properties from words that sound-symbolically represent hardness in Japanese. We prepared Japanese sound-symbolic words of which novelty was manipulated by a genetic algorithm (GA). Japanese speakers in Japan and English speakers in both Singapore and the United States rated these words based on surface texture properties (hardness, warmness, and roughness), as well as familiarity. The results show that hardness-related words were rated as harder and rougher than softness-related words, regardless of novelty and countries. Multivariate analyses of the ratings classified the hardness-related words along the hardness-softness dimension at over 80% accuracy, regardless of country. Multiple regression analyses revealed that the number of speech sounds /g/ and /k/ predicted the ratings of the surface texture properties in non-Japanese countries, suggesting a systematic relationship between phonetic features of a word and perceptual quality represented by the word across culturally and linguistically diverse samples.

## Introduction

A word’s acoustic features and semantics are assumed to be arbitrarily associated in modern linguistics (de Saussure, 1983). This assumption is supported by the fact that different languages employ different sounds to express the same concept. However, it is also known that a non-arbitrary association between sound and meaning, sound symbolism, is present in certain words (Köhler, 1929, 1947; Sapir, 1929; Ramachandran and Hubbard, 2001; Maurer et al., 2006; Westbury et al., 2018). Despite the increasing number of studies on this topic, the nature of sound symbolism is still not fully understood. In the present study, we focused our investigation on the nature of sound symbolism related to surface texture properties.

While different types of sound symbolism are present (e.g., Hinton et al., 1994; Nuckolls, 1999; Cuskley and Kirby, 2013; Lockwood and Dingemanse, 2015), it is generally agreed that sound symbolism can be universal; a certain sound-meaning correspondence in one language can be identified not only by speakers of that language but also by speakers of other languages (Cuskley and Kirby, 2013; Imai and Kita, 2014). One well-known example is the sound symbolism of the shape and size of an object. Specifically, English speakers show high agreement in judging a word form like “Mal” to be a better match for a larger object than “Mil” (Sapir, 1929). Curvy-line drawings are preferentially matched with nonsense words such as “Baluba”/“Maluma” (Köhler, 1929) and “Bouba” (Ramachandran and Hubbard, 2001), whereas an angular shape is preferentially matched with “Takete” (Köhler, 1929) and “Kiki” (Ramachandran and Hubbard, 2001). These systematic relationships between words and shapes/sizes have been replicated cross-lingually and cross-culturally (Davis, 1961; Tarte, 1974; Ramachandran and Hubbard, 2001; Bremner et al., 2013). For instance, the majority of the Himba people with no written language and very minimal exposure to the Western culture matches “Bouba” and “Kiki” to round and angular shapes, respectively (Bremner et al., 2013). These particular sound–shape correspondences appear to be universal despite some exceptions (Rogers and Ross, 1975; Shang and Styles, 2017; Styles and Gawne, 2017).

Although previous studies have demonstrated the universality of sound symbolism regarding shapes and sizes, it is not well understood whether there are systematic relationships between sounds and other physical properties, such as hardness and roughness, of objects. Tangible object properties are categorized into macro-geometric (shapes and sizes) and material properties (roughness and softness; Lederman and Klatzky, 1997). The perception of macro-geometric properties, including shape, orientation, and location, relies on some form of a spatial reference system (spatial coding; Lederman and Klatzky, 1997). Conversely, material properties, including roughness, softness, and warmness, are perceived as intensity (intensity coding; Lederman and Klatzky, 1997). Based on previous studies (e.g., Hollins et al., 1993, 2000), Okamoto et al. (2012) proposed that surface roughness, softness, and warmness are highly prominent perceptual dimensions of the surface of an object (surface texture).

A few previous studies have examined the nature of sound symbolism as it relates to material properties (Sakamoto and Watanabe, 2017, 2018; Hamilton-Fletcher et al., 2018). Hamilton-Fletcher et al. (2018) investigated the effect of visual deprivation on the auditory-tactile relationship between a sound pitch and object properties. Among the surface texture dimensions, high-pitch sounds tend to be associated with softer objects, although the effect is stronger for the blind than for the sighted. In another line of studies, researchers employed Japanese sound-symbolic words that express the impressions of material properties (Sakamoto and Watanabe, 2017, 2018). One of these studies demonstrated that two main principal components accounted for the sound-symbolic words in expressing material properties (Sakamoto and Watanabe, 2017), where roughness, hardness, and warmness were largely explained by one principal component, indicating that sound-symbolic words for expressing surface textures are largely related. Moreover, Sakamoto and Watanabe (2018) demonstrated the existence of systematic relationships between sounds of Japanese words and the dimensions of texture perception. For instance, the sounds /b/+/o/ and /g/+/o/ were employed to express roughness and hardness, whereas /p/+/e/ was employed to express softness and smoothness. Collectively, these findings indicate the possibility of systematic relationships between sounds and perceptual dimensions of texture. Therefore, we asked the following question: is there a cross-cultural and cross-linguistic agreement regarding this kind of sound-to-texture relationship? To the best of our knowledge, the cross-cultural agreement regarding sound symbolism for surface textures has not been examined to this date.

In the present study, we investigated the congruence of judgments for Japanese sound-symbolic words that express properties of surface texture, in Japan and two non-Japanese populations (Singapore and United States). The groups of subjects rated the sound-symbolic words regarding the major perceptual dimensions of surface texture (roughness, softness, and warmness) and reported their familiarity with the words. To control for the effect of familiarity with Japanese words, we generated Japanese sound-symbolic pseudowords by using the system that combines a genetic algorithm (GA) with the automatic estimation of perceived object properties (Shimizu et al., 2015; Doizaki et al., 2017). We hypothesized that subjects from two non-Japanese populations would correctly infer the surface texture properties from Japanese words that sound-symbolically represent either hardness or softness. Moreover, we predicted that there would be systematic relationships between phonetic features of words and the resulting judgment of texture properties across the three different populations.

## Materials and Methods

### Subjects

Data were collected from three countries: the United States (US), Singapore, and Japan (Table 1). The Japanese and Singaporean subjects participated in an offline experiment in which they filled a paper form. The subjects in the US participated in an online experiment. The study protocol was reviewed and approved by the local Ethics Committee at Nanyang Technological University (NTU), Singapore (PSY-IRB-2019-038 and IRB-2020-10-028-01), and the National Institute for Physiological Sciences, Japan (14A045 and 15A030).

**Table 1:** Eligible subjects for the analysis.

	Japanese	Singaporean	United States	
			Pilot	Main sample
Males	17	10	1	11
Females	15	12	20	12
Total	32	22	21	23

#### United States Subjects

To establish a sample size before performing the main experiment, 23 students were recruited from Lasell University in the US for a Qualtrics pilot of the study for which they received five extra credits. Two subjects from this sample were excluded for failing the attention checks, reporting proficiency in the Japanese language, or presenting repeated responses, thus leaving 21 in the pilot sample. The sample size estimation employing G^*^ Power (Faul et al., 2009) indicated that more than 17 subjects were required for alpha = 0.05 and power = 0.95 (dz = 0.84, one-tailed paired *t*-test on the difference of hardness rating). Thus, the results justified the sample size that was utilized in the main experiments.

For the main study, 51 subjects were recruited from Amazon Mechanical Turk (Mturk), where they received 10 USD for participating. Twenty-eight of them were excluded from this sample because they failed the attention-check questions, reported proficiency in the Japanese language, or presented repeated responses; thus, only 23 subjects were obtained from this sample. All the remaining subjects confirmed that they were native English speakers. The mean age of the subjects across the two US samples was 22.6 years (Range = 18–28).

#### Singaporean Subjects

Thirty Singaporeans were recruited from NTU. As described below, eight subjects were excluded from the analysis because they were proficient in Japanese, learning Japanese, or familiar with Japanese words. After the exclusions, the study involved 22 Singaporeans (10 males and 12 females). The mean age of the Singaporean subjects was 22.6 years (Range = 21–25). All the participants confirmed that they were native English speakers.

#### Japanese Subjects

Thirty-two Japanese subjects (17 males and 15 females) were recruited from a previous study by Kitada et al. (2021a) and performed the activity described here as a separate study. The mean age of the Japanese subjects was 23.3 years (Range = 18–35).

### Sound-Symbolic Words

The present study investigated the ratings of 60 sound-symbolic stimuli, which had been previously employed in a functional MRI study of Japanese subjects (Kitada et al., 2021a). The stimuli consisted of 24 conventional Japanese sound-symbolic words (12 “hard”; 12 “soft”), 24 novel sound-symbolic pseudowords aligned with the Japanese patterns (12 “hard”; 12 “soft”), and 12 non-sound-symbolic pseudowords. Table 2 shows that many Japanese sound-symbolic words contain a “core sound,” which conveys the basic meaning of the expression (Inose, 2008). The 24 sound-symbolic words were selected from a Japanese dictionary comprising onomatopoeia: 12 words indicated softness and 12 indicated hardness (Ono, 2007). The 24 novel words were generated as follows: GA generated 300 possible sound-symbolic words (Shimizu et al., 2015; Doizaki et al., 2017) comprising four to six Japanese characters in the GA-generated stimulus set. These GA-generated sound-symbolic pseudowords were examined by 12 Japanese speakers who did not participate in the main study. Twenty-four GA-generated pseudowords, which demonstrated the strongest congruence with the softness (12 pseudowords) and hardness, (12 pseudowords) were selected. To generate the 12 non-sound-symbolic pseudowords, combinations of four Japanese characters were arranged in a pseudo-randomized order. Finally, to check the attention of the participants during the online experiments, six English words (e.g., “clang”) were employed for attention checks for the US subjects (Section “Experimental Procedure”). The words were presented to the Japanese and non-Japanese subjects in the Japanese Hiragana script and standard letters of the Roman alphabet, respectively.

**Table 2:** Words employed in the study.

Conventional sound-symbolic		Novel sound-symbolic		Novel non-sound-symbolic
“Soft”	“Hard”	“Soft”	“Hard”	
Funwaka (ふんわか)	Gachigachi (がちがち)	Munumunu (むぬむぬ)	Kadakada (かだかだ)	Tebahore (てばほれ)
Poyapoya (ぽやぽや)	Gacchingacchin (がっちんがっちん)	Munamuna (むなむな)	Gukuguku (ぐくぐく)	Jizasaki (じざさき)
Powapowa (ぽわぽわ)	Kachinkachin (かちかち)	Yapuyapu (やぷやぷ)	Godogodo (ごどごど)	Ruwagiku (るわぎく)
Fuwari (ふわり)	Gachingachin (がちんがちん)	Payupayu (ぱゆぱゆ)	Gokogoko (ごこごこ)	Wakosatsu (わこさつ)
Hoyahoya (ほやほや)	Kochikochi (こちこち)	Myunomyuno (みゅのみゅの)	Gukaguka (ぐかぐか)	Kosochifu (こちそふ)
Fukafuka (ふかふか)	Gorigori (ごりごり)	Fuyufuyu (ふゆふゆ)	Kagukagu (かぐかぐ)	Machijiya (まちじや)
Pafupafu (ぱふぱふ)	Gichigichi (ぎちぎち)	Fubafuba (ふばふば)	Kakekake (かけかけ)	Buranebo (ぶらねぼ)
Funyafunya (ふにゃふにゃ)	Bakibaki (ばきばき)	Funofuno (ふのふの)	Gukoguko (ぐこぐこ)	Gakigonu (ごきごぬ)
Puyopuyo (ぷよぷよ)	Kachinkochin (かちんこちん)	Pohapoha	Gaigai (がいがい)	Iapeso (いあぺそ)
Fuwafuwa (ふわふわ)	Kachikachi (かちかち)	Myofumyofu (みょふみょふ)	Kogukogu (こぐこぐ)	Nibimuse (びにむせ)
Yuruyuru (ゆるゆる)	Kochinkochin (こちんこちん)	Punopuno (ぷのぷの)	Katokato (かとかと)	Fugusau (ふぐさう)
Kunyakunya (くにゃくにゃ)	Garigari (がりがり)	Bumyabumya (ぶみゃぶみゃ)	Gotgot (ごっごっ)	Shibaroto (しばろと)

*The Japanese Hiragana scripts were presented only to the Japanese group. The words were presented in the Roman script to the non-Japanese group.*

### Experimental Design

In each subject group, we adopted two within-subject factors in which sound symbolism (two levels: softness and hardness) and novelty (two levels: conventional and novel words) were manipulated.

#### Experimental Procedure

The Japanese and Singaporean subjects participated in the offline experiment in which they completed a paper form. The subjects in the US sample performed the task online employing Google forms (pilot) and Qualtrics (main sample). The Japanese and Singaporean subjects completed the test within 30 min. The mean duration of the experiments for Mturk (US) was 27 min.

The subjects first completed the Japanese proficiency check, which was adapted from the Self-Evaluation List in the Official Worldwide Japanese-Language Proficiency Test (2012).^1^ The survey was aimed at measuring the subjects’ self-rated Japanese proficiency and controlling for the Japanese proficiency. The adapted survey included 45 questions, consisting of the following five subscales: (1) “Exposure,” (2) “Spoken,” (3) “Listening,” (4) “Reading,” and (5) “Writing.” The Subjects were required to rate their agreements with statements on a 7-point Likert scale (e.g., “I can understand movies in standard Japanese:” 1 = Strongly disagree, 7 = Strongly agree). The mean responses of each subject within each subscale of the Japanese-language check were computed.

Thereafter, the subjects proceeded to the “Experimental Questionnaire,” which consisted of stimuli that were listed in a pseudo-randomized order, and they were required to rate each word on the (1) softness–hardness, (2) coldness–warmness, and (3) smoothness–roughness tactile material dimensions, as well as evaluate the words for (4) familiarity. The ratings ranged from 0 (very soft, very cold, very smooth, and very unfamiliar) to 10 (very hard, very hot, very rough, and very familiar). For the online subjects, the attention-check questions were incorporated throughout the questionnaire, where the subjects were required to select a specified rating (0, 5, or 10) for one of six English (“Bang,” “Bam,” “Clang,” “Clink,” “Crash,” and “Fwoosh”) word that appeared. Approximately 9% of the words (6 out of 66) were for attention checks.

### Data Analysis

Different analyses were performed to test the two following hypotheses: (1) subjects would exhibit different congruence patterns for “soft” versus “hard” words regardless of their language backgrounds, although the non-Japanese subjects would not distinguish between the novel pseudowords and real Japanese sound-symbolic words and (2) specific speech sounds would be associated with the softness–hardness congruence dimensions. The IBM SPSS Statistics (version 25.0, IBM Corp., Armonk, NY, United States), MATLAB (2020a, MathWorks, Natick, MA, United States), and R packages were employed for the subsequent analyses.

#### Data Exclusions

To minimize any influence of Japanese proficiency on the non-Japanese samples, the Singaporean and US-based Mturk subjects were excluded *via* the following three criteria: first, the subjects whose mean ratings exceeded a score of 3.5 out of 7 in two or more categories of the Japanese proficiency check were excluded from the analysis. Second, the subjects who indicated Japanese as one of their “other languages” were also excluded. Third, since some of the subjects might have been familiar with Japanese sound-symbolic words without explicitly learning Japanese, we excluded some subjects if their mean familiarity ratings for conventional words were 5 or above or if they rated conventional words as more familiar than the novel words by 1 or higher points on the familiarity scale. In addition to these language criteria, the US-based Mturk subjects were excluded for failing to present correct responses to one or more attention-check questions.

#### Analyses of the Primary Hypothesis

We predicted that the Japanese- and non-Japanese-speaking subjects would rate stimulus words as “softer” if they conformed to the Japanese sound symbolism patterns for “soft” words and vice versa for “hard” ones. We also predicted that the Japanese subjects would exhibit stronger congruence effects of conventional sound-symbolic words than for novel GA-generated ones, whereas the non-Japanese speakers would not exhibit this distinction. Univariate analyses were performed within each group to compare the ratings of the four types of sound-symbolic words (conventional “hard,” conventional “soft,” novel “hard,” and novel “soft” words). The pseudowords were included during data visualizations to avail a context for rating the non-sound-symbolic balderdash. In the univariate analyses, we performed conventional analyses with ANOVA [sound symbolism (two levels: softness and hardness) × novelty (two levels: conventional and novel words)].

Next, multivariate analyses that are analogous to the functional MRI data analyses (Kriegeskorte et al., 2008; Ito et al., 2019) were performed to analyze the relationship between the sound-symbolic words. More specifically, we computed the dissimilarities between the words by calculating the pairwise Euclidean distance between the ratings of words in the four dimensions (i.e., hardness, warmness, roughness, and familiarity). Thereafter, the classical multidimensional scaling (MDS) analysis of the group-mean data was performed to visualize the relationship between the words. Further, classification analysis was performed to examine the extent to which the data contained information that would differentiate the “soft” words from the “hard” ones. The mean ratings of the words were randomly separated into four subsets, each containing the data of the same number of words (three words for each category × four chunks). A linear support vector machine (SVM, MATLAB’s SVM) was trained on three subsets of the data and employed to predict the softness–hardness of the words in the remaining subset (25% of the data). The accuracy of the attempted classification was recorded, and the process was repeated four times with a different subset as the test data for the leave-one-run-out cross-validation. In a separate analysis, the same procedure was performed to examine whether the data contained information for classifying each word as conventional or novel.

#### Analyses of the Second Hypothesis

To test the second hypothesis that specific speech sounds would be associated with the softness–hardness congruence dimensions, multiple linear regression analyses were performed. One of the dimensions in the multidimensional scaling (MDS) solution was employed as a dependent variable, while the frequencies of the speech sounds (International Phonetic Alphabet, IPA) in each word were treated as independent predictors. More specifically, the number of times that each speech sound was utilized in each word was counted; for example, “Godogodo” comprises of speech sounds /g/, /o/, and /d/ (i.e., 2^*^/g/ + 4^*^/o/ + 2^*^/d/) compared to “Gokogoko,” which comprises speech sounds /g/, /o/, and /k/ (i.e., 2^*^/g/ + 4^*^/o/+ 2^*^/k/). In the analysis reported here, all the speech sounds, except the geminates in the sound-symbolic words, were treated as regressors thus resulting in 19 regressors (Table 3).^2^ The maximum variance inflation factor (VIF) of each pair of regressors was 2.5, which corresponds to the conservative threshold for collinearity (Johnston et al., 2018). All the regressors and dependent variables were standardized before the analyses.

**Table 3:** Summary of the regression analyses of the three groups.

Variable	Japanese			Singaporean			United States		
	*β*	*t*	P_FWE_	*β*	*t*	P_FWE_	*β*	*t*	P_FWE_
Constant	0.00^†^	0.00^†^	1	0.00^†^	0.00^†^	1	0.00^†^	0.00^†^	1
/a/	−0.13	−0.63	1	−0.30	−1.29	0.618	−0.51	−2.19	0.111
/b/	0.13	1.78	0.257	0.15	2.00	0.167	0.16	2.06	0.147
/tʃ/ = ch	**0.32**	**2.87**	**0.023**	**0.36**	**2.97**	**0.018**	**0.35**	**2.87**	**0.023**
/d/	0.11	1.35	0.568	0.12	1.38	0.533	**0.23**	**2.71**	**0.034**
/e/	−0.05	−0.55	1	−0.13	−1.45	0.477	**−0.29**	**−3.21**	**0.010**
/f/	−0.09	−0.94	1	−0.04	−0.38	1	**0.35**	**3.30**	**0.008**
**/g/**	**0.38**	**3.21**	**0.010**	**0.52**	**4.10**	**0.001**	**0.76**	**5.91**	**0.000** ^*^
/h/	−0.05	−0.56	1	0.07	0.81	1	0.15	1.74	0.279
/i/	0.02	0.11	1	−0.44	−2.08	0.141	−0.53	−2.46	0.061
**/k/**	0.22	1.72	0.292	**0.59**	**4.38**	**0.000** ^*^	**1.20**	**8.83**	**0.000** ^*^
/m/	−0.02	−0.27	1	−0.02	−0.19	2.542	**0.30**	**3.21**	**0.010**
/n/	−0.02	−0.20	1	0.15	1.72	0.290	**0.24**	**2.81**	**0.027**
/o/	−0.14	−0.58	1	−0.51	−2.00	0.166	−**0.66**	−**2.58**	**0.046**
/p/	−0.04	−0.30	1	−0.01	−0.09	1	**0.38**	**2.98**	**0.018**
/r/	0.13	1.63	0.343	0.00^†^	0.04	1	0.20	2.44	0.064
/t/	0.04	0.53	1	0.14	1.93	0.193	0.12	1.64	0.334
/u/	−0.29	−1.24	0.675	**−0.71**	**−2.82**	**0.026**	**−0.78**	**−3.05**	**0.015**
/w/	−0.12	−1.61	0.354	0.06	0.72	1	0.09	1.13	0.802
/j/ = y	−0.13	−1.49	0.441	0.16	1.70	0.302	0.21	2.26	0.096

* *p < 0.0005*.

† *value < 0.005;*

*P_FWE_ indicates the Bonferroni-corrected value of p for the multiple comparisons across the three groups (value of p times 3). The bold letters indicate the significant effects. The signs aligned across the groups for consistency*.

## Results

### Univariate Analyses

To test the hypothesis that subjects would rate the conventional and novel symbolic words in line with Japanese texture associations, univariate analyses were performed for comparing the ratings between the four types of sound-symbolic words. A two-way repeated-measures ANOVA (two levels of sound symbolism × two levels of novelty) on each rating dimension (hardness, warmness, roughness, and familiarity) was performed for each group. Subsequently, paired t-tests were conducted employing the Bonferroni correction if there was any significant interaction. All the mean values and results of ANOVA are available in the Supplementary Tables 1–6.

#### Japanese Group

For each rating scale of interest (hardness, warmness, roughness, and familiarity), we conducted a two-way ANOVA to investigate the effects of the sound-symbolic category (“soft” and “hard” words) and novelty (conventional and novel words) on a subject’s rating. The non-sound-symbolic pseudowords were excluded from these analyses, although they were used a reference for visualizing the rating behavior.

**Figure 1:**
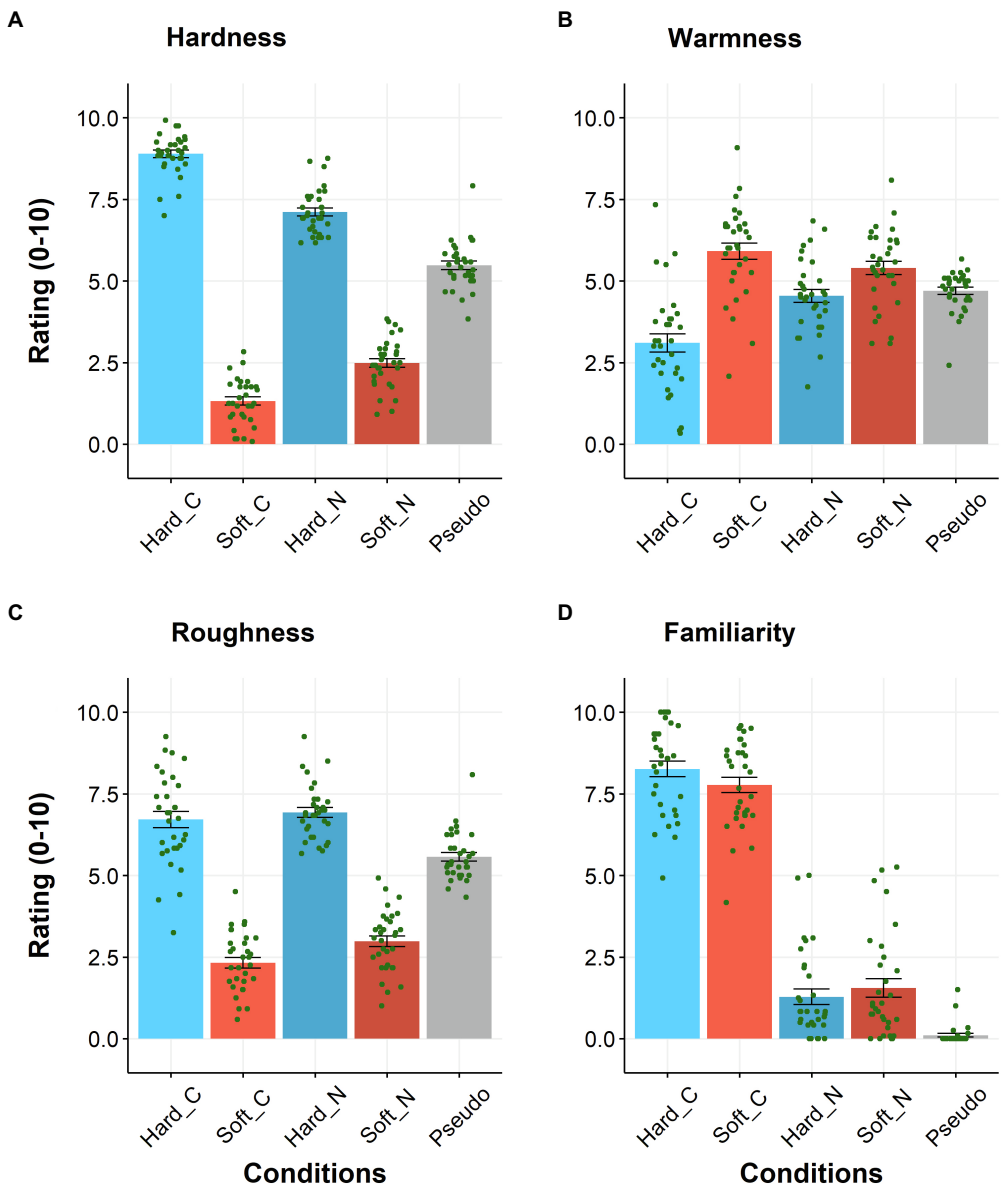
Japanese subjects’ ratings for hardness **(A)**, warmness **(B)**, roughness **(C)**, and familiarity **(D)**. Hard_C, Soft_C, Hard_N, Soft_N, and Pseudo indicate conventional “hard,” conventional “soft,” novel “hard,” and novel “soft” words, as well as non-sound-symbolic pseudowords, respectively. *N* = 32. Error bars indicate the standard errors of the mean (SEM), and each dot indicates each subject’s data.

Figure 1A shows the patterns of the hardness ratings. We observed the significant main effects of sound symbolism [*F*(1, 31) = 1054.85, *p* < 0.001, $\eta_{p}^{2}$ = 0.97] and novelty [*F*(1, 31) = 16.82, *p* < 0.001, $\eta_{p}^{2}$ = 0.35]. In addition, we also observed a significant interaction in which the difference in ratings was larger for the conventional words than for the novel ones [*F*(1, 31) = 251.40, *p* < 0.001, $\eta_{p}^{2}$ = 0.89]. The paired *t*-tests of the hardness ratings (with the Bonferroni correction) confirmed that the “hard” words produced higher hardness ratings than the “soft” words regardless of the novelty [*t*(31) = 35.97, *p* < 0.001, dz = 6.36 for the conventional words; *t*(31) = 22.19, *p* < 0.001, dz = 3.92 for the novel words].

Figure 1B shows the patterns of the warmness ratings. We observed the significant main effects of sound symbolism [*F*(1, 31) = 22.11, *p* < 0.001, $\eta_{p}^{2}$ = 0.42] and novelty [*F*(1, 31) = 17.95, *p* < 0.001, $\eta_{p}^{2}$ = 0.37]. Additionally, a significant interaction was observed in which the difference in ratings (“hard” and “soft” words) was larger for the conventional words than for the novel ones [*F*(1, 31) = 44.06, *p* < 0.001, $\eta_{p}^{2}$ = 0.59]. The paired t-tests of the hardness ratings (with the Bonferroni correction) confirmed that the “soft” words produced higher ratings than the “hard” ones regardless of the novelty [*t*(31) = 5.80, *p* < 0.001, dz = 1.03 for conventional words; *t*(31) = 2.55, *p* = 0.032, dz = 0.45 for the novel words].

Figure 1C shows the patterns of the roughness ratings. There were significant main effects of sound symbolism, where the ratings for the “hard” words were higher than those for the “soft” words [*F*(1, 31) = 306.41, *p* < 0.001, $\eta_{p}^{2}$ = 0.91] and novelty, where the ratings for the novel words were higher than those for the conventional words [*F*(1, 31) = 15.53, *p* < 0.001, $\eta_{p}^{2}$ = 0.33], with no significant interaction (*p* = 0.21).

Figure 1D shows the familiarity ratings. A significant main effect of novelty [*F*(1, 31) = 888.76, *p* < 0.001, $\eta_{p}^{2}$ = 0.97] in which the conventional words scored higher ratings than the novel ones was observed. However, we also observed a significant interaction in which the difference in the ratings was in the opposite direction for the “hard” and “soft” words [*F*(1, 31) = 10.26, *p* = 0.003, $\eta_{p}^{2}$ = 0.25]. No main effect of sound symbolism was observed (*p* > 0.4). The paired t-tests (with the Bonferroni correction) confirmed that the familiarity ratings were higher for the conventional words than for the novel ones regardless of the types of sound-symbolic words [*t*(31) = 25.45, *p* < 0.001, dz = 4.50 for the “soft” words; *t*(31) = 26.95, *p* < 0.001, dz = 4.76 for the “hard” words].

Collectively, we confirmed that the conventional and novel words generated the ratings of hardness and familiarity along the expected direction among the Japanese subjects. Additionally, the categorization of words as “hard” or “soft” also guided the ratings of the warmness (hard = cold) and roughness (hard = rough) dimensions.

#### Singapore Group

**Figure 2:**
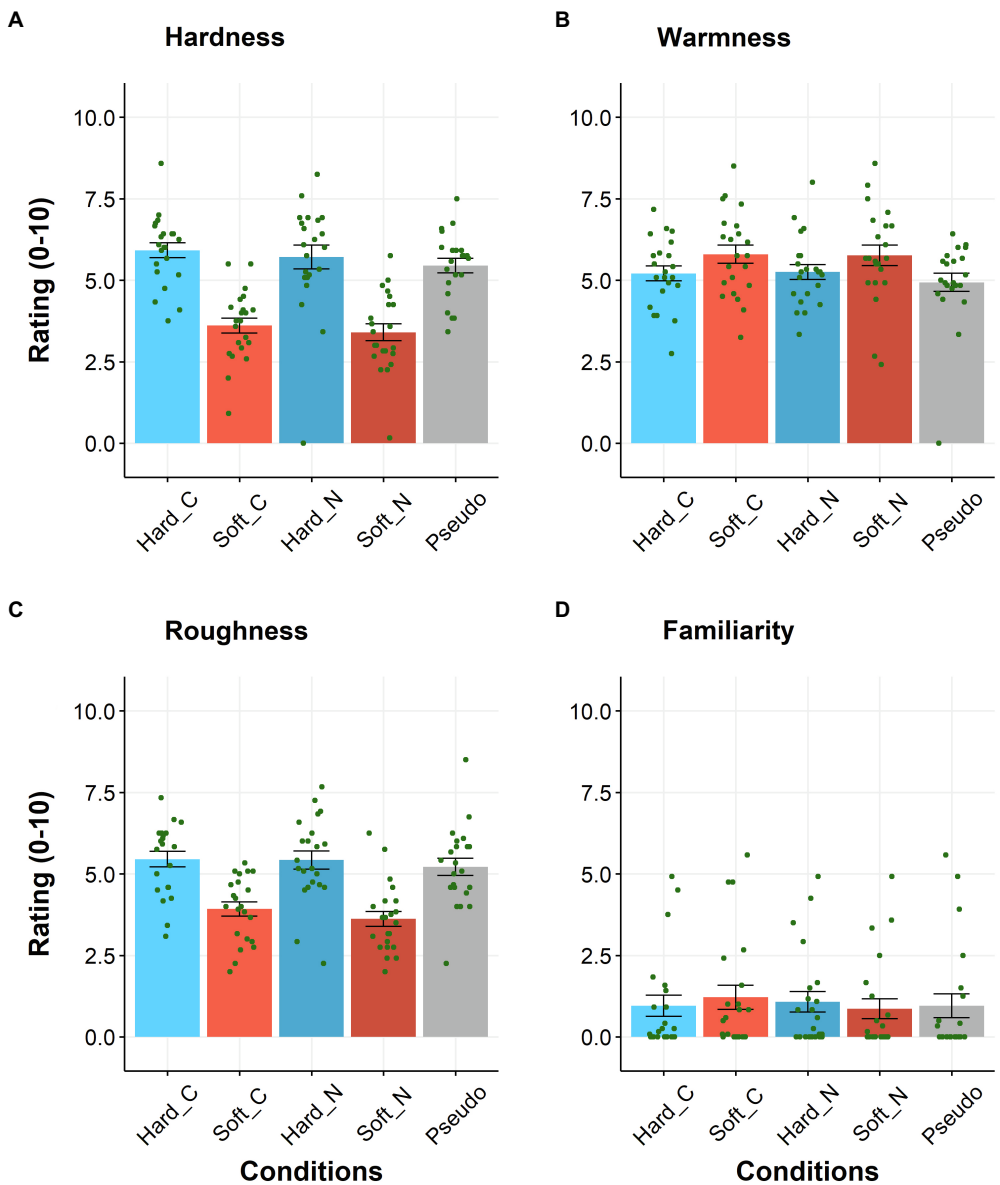
Singaporean subjects’ ratings for hardness **(A)**, warmness **(B)**, roughness **(C)**, and familiarity **(D)**. Hard_C, Soft_C, Hard_N, Soft_N, and Pseudo indicate conventional “hard,” conventional “soft,” novel “hard,” and novel “soft” words, as well as non-sound-symbolic pseudowords, respectively. *N* = 22. Error bars indicate SEM, and each dot indicates each subject’s data.

After screening the subjects based on their Japanese proficiency and familiarity with Japanese words, the remaining 22 subjects were analyzed. Their hardness and roughness ratings exhibited highly similar patterns; the rating for the “hard” words was higher than that for the “soft” words (Figures 2A,C). The two-way repeated-measures ANOVA of the hardness rating revealed the significant main effect of sound symbolism [*F*(1, 21) = 64.91, *p* < 0.001, $\eta_{p}^{2}$ = 0.76]. Similarly, the same ANOVA of the roughness rating revealed the significant main effect of sound symbolism [*F*(1, 21) = 29.57, *p* < 0.001, $\eta_{p}^{2}$ = 0.59]. No other significant effect was observed in these ANOVA tests (values of *p* > 0.1). However, the same ANOVA of the warmness rating did not exhibit any significant effect (values of *p* > 0.09, Figure 2B).

#### Does the Familiarity With Japanese Words Explain the Effect of Sound Symbolism Among the Singaporean Subjects?

Figure 2D shows that the mean rating for familiarity was less than 1.5. The two-way repeated-measures ANOVA of the familiarity rating exhibited a significant interaction [*F*(1, 21) = 6.62, *p* = 0.018, $\eta_{p}^{2}$ = 0.24]. The paired t-tests (with the Bonferroni correction) showed that the familiarity ratings for the conventional “soft” words were higher than for the novel “soft” words [*t*(21) = 3.16, *p* = 0.01, dz = 0.67]. Conversely, such a familiarity effect was not observed on the “hard” words (*p* > 0.5). This result indicates that the conventional “soft” words might be slightly more familiar than the novel “soft” ones.

To assess the effect of familiarity, linear regression analyses of the effect of sound symbolism were performed employing the familiarity ratings as the covariates of no interest. If the effect of sound symbolism on the hardness ratings was merely due to the familiarity effect, the parameter estimates (*β*) of the constant terms should not differ from zero. In this analysis, the effect of sound symbolism was calculated by subtracting the ratings of the “soft” words from those of the “hard words” (see the Supplementary Material for calculating covariates). Nevertheless, *β* of the constant terms corresponding to the effect of sound symbolism were significantly greater than zero [*t*(19) = 6.88, *p* < 0.001 for the conventional words; *t*(19) = 4.92, *p* < 0.001 for the novel words]. The same analysis of the roughness ratings also exhibited the same patterns, namely, *β* for the constant terms being significantly higher than zero [*t*(19) = 3.30, *p* = 0.004 for the conventional words; *t*(19) = 2.88, *p* = 0.009 for the novel words]. These findings indicate that sound symbolism affects the hardness and softness perceptions even after adjusting for the familiarity effect.

#### The US Group

**Figure 3:**
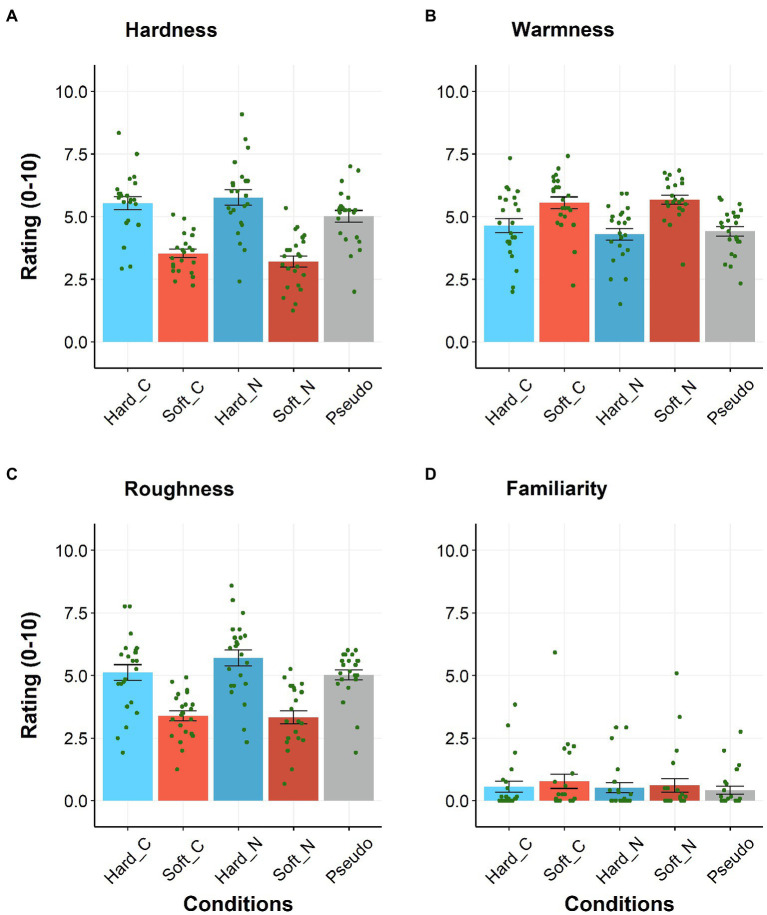
US subjects’ ratings for hardness **(A)**, warmness **(B)**, roughness **(C)**, and familiarity **(D)**. Hard_C, Soft_C, Hard_N, Soft_N, and Pseudo indicate conventional “hard,” conventional “soft,” novel “hard,” and novel “soft” words, as well as non-sound-symbolic pseudowords, respectively. *N* = 23. Error bars indicate SEM, and each dot indicates each subject’s data.

After screening the subjects based on their Japanese proficiency and familiarity with Japanese words, the remaining 23 subjects were analyzed. The hardness and roughness ratings exhibited highly similar patterns in which the rating for the “hard” words was greater than that for the “soft” ones (Figures 3A,C). The two-way repeated-measures ANOVA of the hardness rating revealed the significant main effect of sound symbolism [*F*(1, 22) = 45.40, *p* < 0.001, $\eta_{p}^{2}$ = 0.67], and no other significant effect was observed (values of *p* > 0.08). The same ANOVA of the roughness rating revealed significant main effects of sound symbolism, where the “hard” words produced a higher rating than the “soft” words [*F*(1, 22) = 25.80, *p* < 0.001, $\eta_{p}^{2}$ = 0.54] and novelty, with the novel words producing a higher rating than the conventional words [*F*(1, 22) = 5.59, *p* = 0.027, $\eta_{p}^{2}$ = 0.20]. No significant interaction was observed (*p* = 0.069).

The same ANOVA of the warmness rating revealed significant main effects of sound symbolism in which the “soft” words produced higher warmness ratings than the “hard” words [*F*(1, 22) = 19.55, *p* < 0.001, $\eta_{p}^{2}$ = 0.47; Figure 3B]. No other significant effect was observed (values of *p* > 0.09).

Finally, the same ANOVA of familiarity showed no significant effect (values of *p* > 0.2, Figure 3D). Collectively, the “hard” words produced significantly harder, rougher, and colder ratings than the “soft” ones regardless of novelty of the words.

### Multivariate Analyses

The univariate analyses revealed that the Japanese “hard” sound-symbolic words generated harder and rougher ratings regardless of the linguistic and cultural groups. However, it was unclear whether most of the words in each category contributed to the sound-symbolic effect or whether a few specific words accounted for the effect. To examine the rating patterns for each word, we conducted MDS and classification analyses using a SVM.

#### Dissimilarity Between the Words

**Figure 4:**
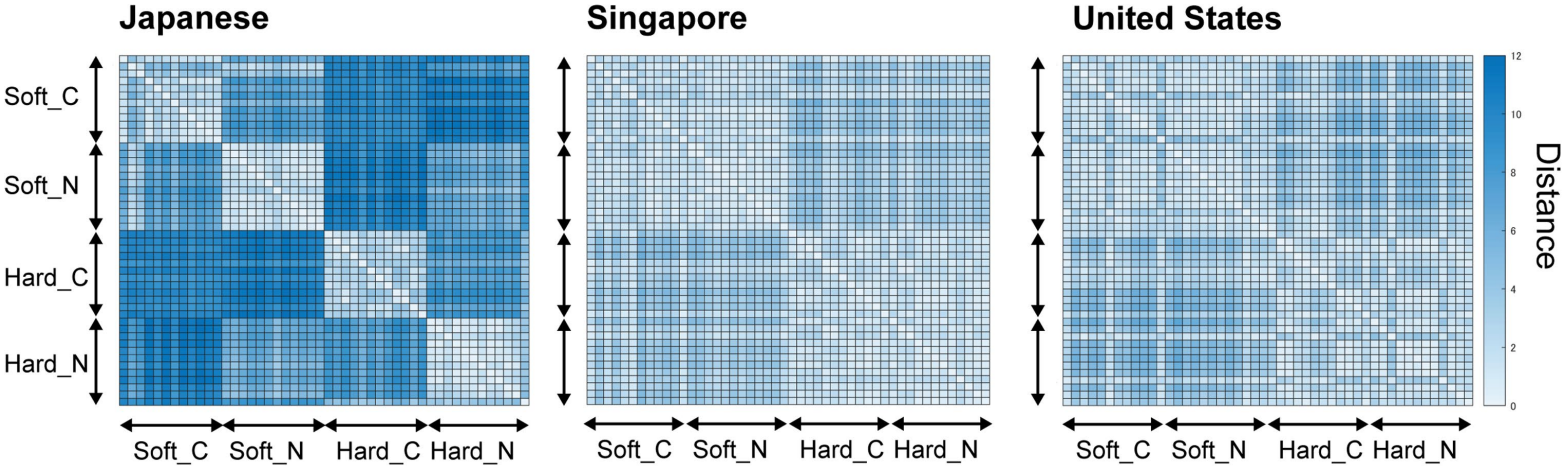
Dissimilarity matrices for the 48 sound-symbolic words. We calculated the Euclidean distance between each pair of words employing the ratings of four dimensions (hardness, warmth, roughness, and familiarity). Horizontal and vertical axes indicate the list of words shown in Table 2. Soft_C, Soft_N, Hard_C, and Hard_N indicate the conventional softness, novel softness (generated by GA), conventional hardness, and novel hardness words, respectively. Each pixel indicates the difference between the distance of the words (the higher the distance, the bluer the pixel looks). The order of the words in each category (from top to bottom) is the same as the order of the words in Table 2. Since the orders of the words were the same along the horizontal and vertical axes, a symmetrical matrix was obtained.

Figure 4 shows the dissimilarity of the group-mean ratings of the Japanese, Singaporean, and US subjects. Each axis of the dissimilarity matrix includes the 48 tested sound-symbolic words, and the white–blue scale in each cell represents the Euclidean distance between the mean of each word and other words. A visual inspection revealed that the dissimilarities within each combination of sound symbolism and novelty, e.g., conventional “soft” words, were smaller than those between the different combinations of sound symbolism and novelty, e.g., conventional “soft” vs. novel “soft” words, in the Japanese subjects. For the Singaporean and US subjects, the distance within the same category of sound symbolism, e.g., “soft” words, is smaller than that between the different types of sound symbolism, e.g., “soft” vs. “hard” words. Conversely, the dissimilarity between the conventional and novel words was as low as the dissimilarities between each combination of the sound symbolism and novelty.

#### MDS

**Figure 5:**
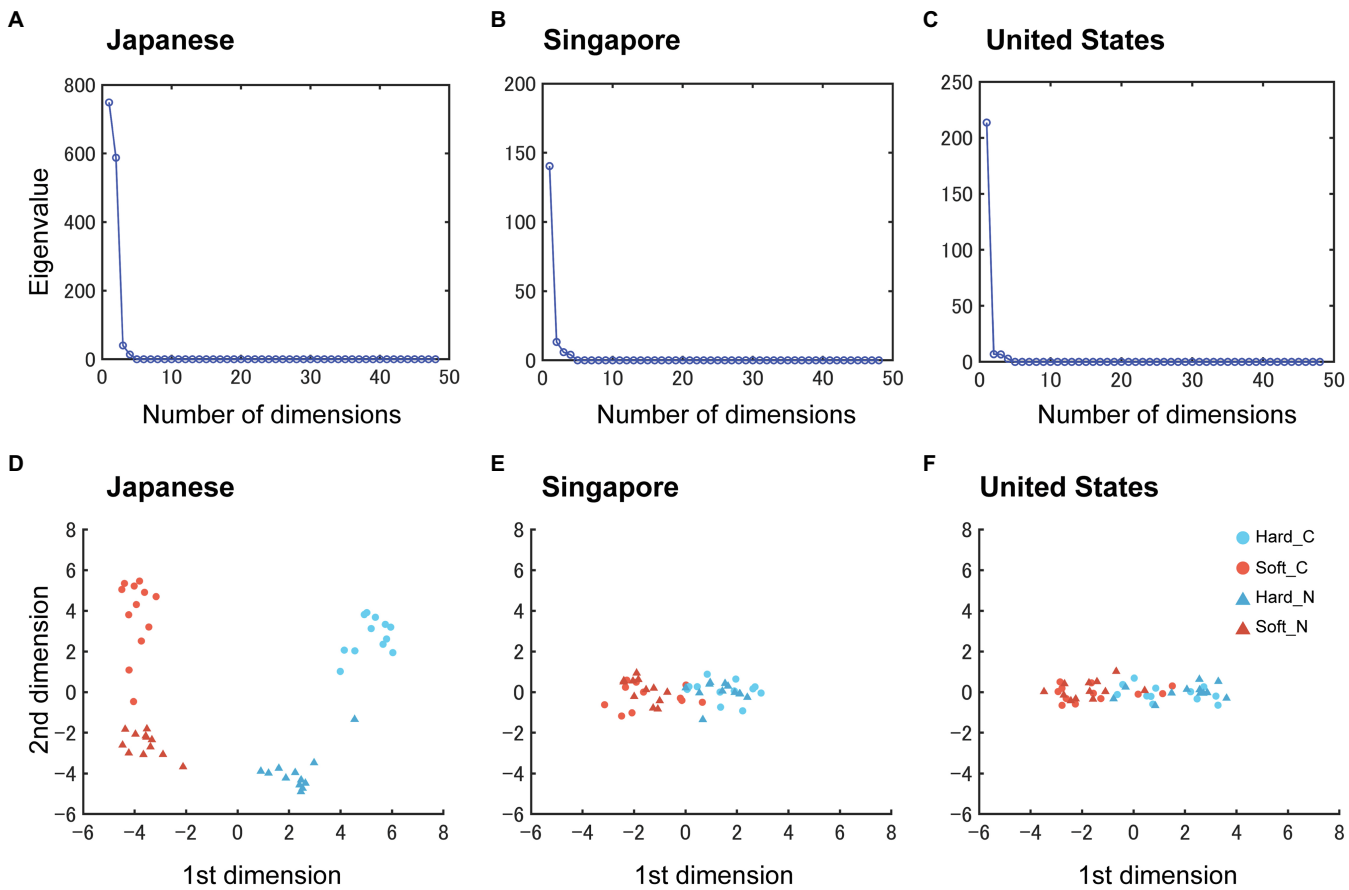
Multidimensional Scaling (MDS) of each country data. **(A–C)**, Scree Plots for the Japanese, Singaporean, and US subjects, respectively. The scales of the *y*-axes in **(B,C)** were adjusted for presentation purposes. **(D–F)**, 2D MDS solutions for the Japanese, Singaporean, and US subjects, respectively. Each dot represents a sound-symbolic word.

MDS was performed to visualize the relationships between the sound-symbolic words. An inspection of the scree plots revealed that two dimensions were sufficient (Figures 5A–C). The visual inspection of the two-dimensional (2D) MDS solutions indicates that the first dimension (the *x*-axis in Figure 5) represents the surface texture properties (softness, warmth, and roughness), whereas the second dimension (the *y*-axis in Figure 5) indicates the novelty of the words. The “soft” words were separately clustered from the “hard” ones in all groups, although the Singapore and US data exhibited a few overlaps (Figures 5D–F). More specifically, the “soft” conventional words “funwaka,” “fukafuka,” and “kunyakunya” were located within the “hard” word cluster (orange dots within the blue cluster) for Singaporean and US subjects. The conventional and novel words appeared to be clustered separately only for Japanese subjects.

#### Classification Analysis Employing SVM

The classification analyses of the subjects’ ratings were performed employing an SVM classifier. The classification accuracy of the “soft” versus “hard” words was 100, 89.6, and 85.4% for the Japanese, Singaporean, and US-based Mturk subjects, respectively [accuracy was well above the chance level (50%) across all groups]. On the other hand, the classification accuracy of the conventional versus novel words (chance level = 50%) was 97.9% for the Japanese subjects but much lower for the non-Japanese groups (39.6 and 54.2% for the Singaporean and US-based Mturk subjects, respectively). These results confirm that the group-mean rating data contained information with which the Singaporean and US subjects classified the words as “hard” or “soft.”

### Multiple Regression

The foregoing multivariate analyses revealed that subjects correctly classified the sound-symbolic words along the hardness-softness dimension in Japanese regardless of their novelty, thereby raising the possibility that specific speech sound patterns might have contributed to the observed sound-to-texture mapping (Nielsen and Rendall, 2011, 2013). The first MDS dimension (the x dimension in Figures 5D–F) separated the softness-related words from the hardness-related ones based on the surface texture. Thus, multiple regression analyses were performed employing the first MDS dimension as the dependent variable and the frequencies of each speech sound (IPA), i.e., 19 in the sound-symbolic words, as the independent variables (regressors).

Table 3 presents the multiple regression analysis results. Across the three groups, the parameter estimates (*β*) for the speech sound /g/ and /ch/ were significantly greater than zero. *β* for /k/were significantly greater than zero in the non-Japanese groups, indicating that these speech sounds were associated with the values in the first dimension of the MDS solutions. As a supplementary analysis, the same analysis was performed employing the hardness ratings as the dependent variables, and the same findings were obtained only for /g/ and /k/ (Supplementary Table 6).

## Discussions

This study presents two main findings. First, the “hard” Japanese sound-symbolic words were rated as harder and rougher than the “soft” ones by the Japanese speakers, as well as non-Japanese speakers who were unfamiliar with the words and Japanese sound symbolism patterns. Additionally, the Japanese speakers exhibited stronger effects of the sound-symbolic congruence than the other groups partly driven by more polarized ratings for conventional Japanese sound-symbolic words than for the GA-generated pseudowords. Contrarily, the subjects in Singapore and the US did not substantially differentiate between the conventional and novel sound-symbolic words, indicating that something about the phonetic forms of the words (or the letters by which they were expressed) accounted for the sound-symbolic effects. The speech sounds /g/ contributed to the increased congruence of a word with surface texture characteristics (hardness, roughness, and warmth) regardless of the culture and language. Moreover, the speech sound /k/ increased the same effect of congruency among the non-Japanese-speaking populations.

We controlled for the subjects’ familiarity with Japanese in several ways. First, the Singaporean and US subjects who exhibited Japanese proficiency were excluded from the study. Second, the subjects whose familiarity ratings were high or differed between the conventional and novel words were excluded from the analysis. Thus, it was unlikely that these subjects distinguished the “soft” words from the “hard” ones through their explicit knowledge of Japanese words.

Previous studies reported that sound symbolism of Japanese words was related to the surface texture (Sakamoto and Watanabe, 2017, 2018). These studies demonstrated the existence of systematic relationships between speech sounds of sound-symbolic words and the dimensions of texture perception among Japanese populations. Another study revealed the effect of visual deprivation on the congruence between pitch and surface texture (Hamilton-Fletcher et al., 2018). However, it was unclear whether the congruence of Japanese words with surface texture characteristics would be similarly observed among the non-Japanese populations. Thus, the current study has extended the previous findings by demonstrating that non-Japanese individuals can distinguish Japanese sound-symbolic words related to hardness-softness and that systematic relationships exist between speech sounds of a word and the perceptual quality (e.g., hardness) of that word across a linguistically diverse sample.

### Cross-Cultural Similarity of Sound Symbolism Regarding the Surface Texture

Subjects in all three groups rated “hard” Japanese words as harder than “soft” words. Multiple regression analyses revealed that the speech sounds “/g/” and “/k/” were associated with “hard” Japanese sound-symbolic words by the non-Japanese groups. This result indicates that specific consonants were related to the perception of hardness in these countries, which is consistent with previous work reporting that some combinations of vowels and consonants, including /g/, could be associated with hardness among the Japanese population (Sakamoto and Watanabe, 2017).

This cross-cultural/lingual similarity in sound-meaning correspondences could be partially explained by the letters employed in the Bouba–Kiki effect. For instance, the angular shape was preferentially associated with Takete (Köhler, 1929, 1947) and Kiki (Ramachandran and Hubbard, 2001), which include the letter “K.” Indeed, the objects must be sufficiently hard to form spiky or angular shapes of real 3D objects; slime cannot form spiky shapes. Thus, the speech sound /g/ might be associated with the angular shapes of objects and their material properties, conveying hard, rough, and angular shapes.

According to the IPA consonant chart, /g/ and /k/ are both categorized as plosive (consonants produced by blocking the airflow, followed by an abrupt release) and velar consonants (articulated *via* a placement of the back part of the tongue against the soft palate). Nielsen and Rendall (2013) demonstrated that plosive consonants and nonrounded vowels were associated with jagged object images when subjects selected specific syllables that best matched the presented images (Nielsen and Rendall, 2013). Considering that the Bouba–Kiki effect could be observed cross-lingually and cross-culturally (Davis, 1961; Tarte, 1974; Ramachandran and Hubbard, 2001; Bremner et al., 2013), it is possible that /g/ was associated with hardness and roughness regardless of the language and country.

The critical difference between /g/ and/k/ is that the Japanese subjects associated hardness with /g/ but not with /k/. Previous studies revealed that the differences in the orthographic forms of words, such as letter shape, could affect sound-meaning correspondences (Cuskley et al., 2017; Turoman and Styles, 2017). We presented different written scripts to the Japanese and non-Japanese groups (Hiragana and alphabets to the Japanese and non-Japanese speakers, respectively). However, the shapes of Hiragana associated with /k/ appear to be as rounded as other Hiragana characters. Moreover, we confirmed that the speech sounds associated with these words were related to hardness in a pilot experiment on Japanese. Thus, it was unlikely that the differences in the shapes of the presented orthographic forms would explain the group differences. Rather, knowledge of Japanese might cause this group-specific effect. For instance, conventional /k/-containing Japanese words, such as “funwaka,” “fukafuka,” and “kunyakunya,” are related to soft objects, whereas /k/ is not included among the novel softness words. Thus, prior knowledge of these Japanese words could mask the non-arbitrary relationship between /k/ and hardness perception. Future studies could further investigate this speculation.

Despite some of the group differences, as discussed, our findings largely agree with those of previous studies demonstrating that some sound-symbolic words in a given language could be understood by speakers of other languages (Cuskley and Kirby, 2013; Imai and Kita, 2014 for review). For instance, Iwasaki and colleagues demonstrated that English speakers without prior knowledge of Japanese and native Japanese speakers similarly judged Japanese sound-symbolic words, such as laughing and walking (Iwasaki et al., 2007a) and pain (Iwasaki et al., 2007b). Moreover, English-speaking adults could learn the definitions of Japanese adjectives, e.g., “akarui” meaning bright, faster when they were paired with their actual meaning than when they were randomly paired (Nygaard et al., 2009). Thus, certain aspects of Japanese words, e.g., ideophones, might be interpreted similarly regardless of the linguistic background. This speculation is consistent with a more recent finding that subjects in diverse cultural/linguistic populations inferred the words *big* and *small* more accurately in more widely spoken languages including Japanese (Lev-Ari et al., 2021).

Contrary to “hard” words, we observed that no single consonant was associated with the perception of softness across the three countries (represented as negative *β* in multiple regression analyses). In previous studies, curvy-line drawings were associated with “Baluba/Malma” (Köhler, 1929) and “Bouba” (Ramachandran and Hubbard, 2001). Dissimilar to the spikey or angular shapes, the curvy ones could be associated with both soft and hard objects (bowling ball), and this might weaken the relationship between softness and letters, such as “B” and “M.” Another account is that the contact with hard surfaces (e.g., knocking on a wooden door) would generate a louder sound than contact with soft surfaces (e.g., squeezing a plush toy). Thus, the fewer chances of soft objects generating loud sounds when compared to hard objects might contribute to the weak relationship between letters and the perception of softness.

### Roughness Associated With “Hard” Words

We observed that subjects rated “hard” Japanese words as rougher across all three groups. There are two accounts regarding the covariation between the perception of hardness and roughness. First, there are fewer objects with surfaces that are concurrently rough and soft, whereas many textile products exhibit simultaneously soft and smooth surfaces (silk blanket). Even with the spatial variations of soft surfaces, e.g., sponge, a contact force would deform the surface of the object such that it would not feel as rough as spatial variations on a hard surface. Thus, it is less frequent to encounter objects exhibiting combined softness and roughness, which might result in stronger association between hardness and roughness. Second, through linguistic descriptions, such as metaphors (Ackerman et al., 2010), physical properties can be linked to mental metaphors (Casasanto and Gijssels, 2015). For instance, roughness and hardness are often used to convey similar meanings, such as difficulty (e.g., having a rough/hard day). Tactile perception of harder and rougher objects can cause less pleasantness than softer and smoother objects (Kitada et al., 2012, 2021b; Pasqualotto et al., 2020). Thus, sound-symbolic words related to hardness might be implicitly associated with roughness.

### Limitations and Future Directions

There are four notable limitations to this study. First, although we minimized the effect of familiarity with Japanese sound-symbolic words, the subjects might have experienced Japanese-related information (e.g., animation) and developed an implicit relationship between speech sounds (/g/) and perception of material properties. Thus, future studies could examine whether the observed effect is generalizable by examining a subject group that is not exposed to Japanese. Second, due to geographical constraints and limited resources, the portion of the study involving US subjects was conducted online, whereas the part involving the Japanese and Singaporean subjects was conducted *via* the pen-and-paper method. Although we anticipate minimal impact from this methodical difference, it would still be preferable to conduct future studies in a standardized and homogeneous manner to improve data reliability. Third, this study only incorporated visual texts of sound-symbolic words without accompanying auditory cues because we intended to remove prosody or pitch that might be associated with material properties. Although consonants, such as /g/ and /k/, are pronounced similarly in all tested countries, the differences in the linguistic backgrounds might have resulted in the different pronunciations of the words. Future studies could also consider including auditory cues with the corresponding visual texts to ensure the validity of the results. Finally, we presented Hiragana and alphabets to the Japanese and non-Japanese speakers, respectively. To control for any influence of orthographic forms, it is important in future studies to use the Roman scripts for the Japanese subjects.

## Conclusion

This study examined the cross-cultural/cross-lingual similarity between Japanese sound-symbolic words and the hardness-softness representations of objects. Our results demonstrate that the Singaporean and US subjects, as well and Japanese subjects, judged “hard” and “soft” words correctly. Particularly, specific letters, e.g., “G,” were associated with objects’ hardness and roughness regardless of the cultural and linguistic backgrounds. This result indicates that some speech sounds contain information that are associated with hardness and softness in different cultural and linguistic backgrounds. This finding contributes to better understanding the nature of sound symbolism, especially non-arbitrary relationship between speech sounds and physical properties of objects.

## Data Availability Statement

The datasets generated and analyzed during this study is available at: https://osf.io/s37fc/.

## Ethics Statement

The studies involving human participants were reviewed and approved by the Local Ethics Committee at Nanyang Technological University (PSY-IRB-2019-038 and IRB-2020-10-028-01) and the National Institute for Physiological Sciences, Japan (14A045 and 15A030). Written informed consent for participation was not required for US subjects in accordance with the national legislation and the institutional requirements.

## Author Contributions

RK and LSW designed the study and analyzed the data. LSW, JK, ZZ, and RK performed the research. JK and MS contributed to the generation of the novel words. LSW, SJS, and RK wrote the paper. All authors contributed to the article and approved the submitted version.

## Funding

This work was supported by a NAP start-up grant from NTU and MEXT/JSPS KAKENHI (Fund for the Promotion of Joint International Research, 20 K23372), Japan, to RK, and a NAP start-up grant from NTU to SJS (04INS000116C430).

## Conflict of Interest

The authors declare that the research was conducted in the absence of any commercial or financial relationships that could be construed as a potential conflict of interest.

## Publisher’s Note

All claims expressed in this article are solely those of the authors and do not necessarily represent those of their affiliated organizations, or those of the publisher, the editors and the reviewers. Any product that may be evaluated in this article, or claim that may be made by its manufacturer, is not guaranteed or endorsed by the publisher.

## Footnotes

^1^ https://www.jlpt.jp/e/about/candolist.html

^2^The precise phonological status of geminates in Japanese is a subject of several debates. Given that only two of the target words contained geminates and that geminates generally occurred at the same voicing and articulation place as the following consonant, they were excluded from the main analysis.
