# Supplementary material for: Japanese Sound-Symbolic Words for Representing the Hardness of an Object Are Judged Similarly by Japanese and English Speakers
Source: Front Psychol. 2022 Mar 15;13:830306. doi: 10.3389/fpsyg.2022.830306 (PMC8965287; doi:10.3389/fpsyg.2022.830306)
Supplement: Supplementary file 1 [file Data_Sheet_1.docx]

Supplementary Material

**Japanese sound-symbolic words for representing the hardness of an object are judged similarly by Japanese and English speakers**

# Supplementary Method

To tease apart the effect of familiarity from the effect of sound symbolism, we also conducted linear regression analyses with familiarity ratings as covariates. We calculated the effect of sound symbolism on hardness for each subject by subtracting mean hardness ratings of conventional “soft” words from mean hardness ratings of conventional “hard” words. This sound-symbolic effect on the hardness-softness dimension was used as a dependent variable. We used the two types of familiar ratings as covariates. First, we calculated the average of familiarity ratings for the conventional words to represent the overall familiarity with Japanese words. Second, we computed the difference of familiarity ratings between “hard” and “soft” words (of conventional words) to represent the effect of sound symbolism on familiarity. The same analysis was conducted for hardness ratings of novel words and for other ratings in which the effect of sound symbolism was observed.

# Supplementary Tables

**Supplementary Table 1.** Mean Ratings for Each Subject Group

|  | Soft_C | Hard_C | Soft_N | Hard_N | Pseudo |
| --- | --- | --- | --- | --- | --- |
| **Japan** |  |  |  |  |  |
| **Hardness** | | | | | |
| Mean | 1.32 | 8.89 | 2.48 | 7.11 | 5.48 |
| SEM | 0.40 | 0.29 | 0.51 | 0.33 | 0.25 |
| **Warmness** |  | |  |  |  |
| Mean | 5.91 | 3.10 | 5.40 | 4.54 | 4.70 |
| SEM | 0.75 | 0.96 | 0.51 | 0.36 | 0.08 |
| **Roughness** |  | |  |  |  |
| Mean | 2.32 | 6.71 | 2.98 | 6.93 | 5.57 |
| SEM | 0.42 | 0.59 | 0.52 | 0.29 | 0.46 |
| **Familiarity** |  | |  |  |  |
| Mean | 7.77 | 8.26 | 1.55 | 1.28 | 0.10 |
| SEM | 0.64 | 0.82 | 1.17 | 0.65 | 0.06 |
| **Singapore** | | | | | |
| **Hardness** | | | | | |
| Mean | 3.61 | 5.92 | 3.40 | 5.71 | 5.45 |
| SEM | 0.23 | 0.23 | 0.26 | 0.36 | 0.22 |
| **Warmness** | | | | | |
| Mean | 5.80 | 5.20 | 5.77 | 5.25 | 4.93 |
| SEM | 0.28 | 0.23 | 0.31 | 0.23 | 0.28 |
| **Roughness** | | | | | |
| Mean | 3.92 | 5.45 | 3.62 | 5.42 | 5.21 |
| SEM | 0.21 | 0.24 | 0.23 | 0.28 | 0.26 |
| **Familiarity** | | | | | |
| Mean | 1.21 | 0.95 | 0.86 | 1.08 | 0.95 |
| SEM | 0.37 | 0.32 | 0.31 | 0.32 | 0.36 |
| **United States** | | | | | |
| **Hardness** | | | | | |
| Mean | 3.53 | 5.53 | 3.21 | 5.75 | 5.01 |
| SEM | 0.16 | 0.26 | 0.22 | 0.31 | 0.23 |
| **Warmness** | | | | | |
| Mean | 5.55 | 4.64 | 5.67 | 4.29 | 4.41 |
| SEM | 0.24 | 0.28 | 0.17 | 0.23 | 0.19 |
| **Roughness** | | | | | |
| Mean | 3.39 | 5.11 | 3.33 | 5.69 | 5.02 |
| SEM | 0.19 | 0.31 | 0.25 | 0.32 | 0.20 |
| **Familiarity** | | | | | |
| Mean | 0.77 | 0.56 | 0.61 | 0.51 | 0.42 |
| SEM | 0.29 | 0.21 | 0.26 | 0.20 | 0.16 |

**Supplementary Table 2.** Mean Ratings for Each Word (Japanese group)

| Words | Type | Hardness | Warmness | Roughness | Familiarity |
| --- | --- | --- | --- | --- | --- |
|  |  |  |  |  |  |
| funwaka | Soft_C | 1.03 | 5.84 | 2.16 | 7.75 |
| poyapoya | Soft_C | 1.97 | 5.72 | 2.41 | 3.53 |
| powapowa | Soft_C | 1.53 | 5.94 | 2.34 | 5.06 |
| fuwari | Soft_C | 1.16 | 5.50 | 1.75 | 9.13 |
| hoyahoya | Soft_C | 2.06 | 7.66 | 3.06 | 7.47 |
| fukafuka | Soft_C | 0.72 | 6.84 | 2.28 | 9.34 |
| pafupafu | Soft_C | 1.44 | 6.09 | 2.97 | 6.63 |
| funyafunya | Soft_C | 0.44 | 5.44 | 1.97 | 8.91 |
| puyopuyo | Soft_C | 1.88 | 4.91 | 2.13 | 8.72 |
| fuwafuwa | Soft_C | 0.94 | 5.84 | 2.53 | 9.50 |
| yuruyuru | Soft_C | 1.59 | 5.66 | 2.00 | 8.91 |
| kunyakunya | Soft_C | 1.09 | 5.50 | 2.28 | 8.28 |
| gachigachi | Hard_C | 9.66 | 3.66 | 7.06 | 8.88 |
| gacchingacchin | Hard_C | 9.75 | 3.25 | 6.47 | 7.91 |
| kachinkachin | Hard_C | 9.19 | 2.63 | 5.38 | 9.22 |
| gachingachin | Hard_C | 9.81 | 2.75 | 6.97 | 7.56 |
| kochikochi | Hard_C | 8.34 | 2.59 | 5.66 | 6.22 |
| gorigori | Hard_C | 8.56 | 3.75 | 8.63 | 8.44 |
| gichigichi | Hard_C | 8.22 | 4.63 | 6.72 | 7.53 |
| bakibaki | Hard_C | 8.47 | 3.72 | 7.50 | 8.75 |
| kachinkochin | Hard_C | 9.69 | 1.81 | 4.94 | 8.94 |
| kachikachi | Hard_C | 9.22 | 2.38 | 5.06 | 9.06 |
| kochinkochin | Hard_C | 7.81 | 4.03 | 7.81 | 7.63 |
| garigari | Hard_C | 8.00 | 2.00 | 8.34 | 9.00 |
| munumunu | Soft_N | 3.13 | 5.81 | 2.63 | 1.78 |
| munamuna | Soft_N | 3.34 | 5.53 | 3.19 | 1.16 |
| yapuyapu | Soft_N | 2.69 | 5.47 | 2.75 | 1.00 |
| payupayu | Soft_N | 1.78 | 5.59 | 2.47 | 2.13 |
| myunomyuno | Soft_N | 2.03 | 5.66 | 2.19 | 1.31 |
| fuyufuyu | Soft_N | 2.06 | 3.91 | 2.91 | 1.78 |
| fubafuba | Soft_N | 3.22 | 5.56 | 5.00 | 0.84 |
| funofuno | Soft_N | 2.09 | 5.38 | 2.53 | 0.97 |
| pohapoha | Soft_N | 2.75 | 6.00 | 3.28 | 1.50 |
| myofumyofu | Soft_N | 2.00 | 5.44 | 2.88 | 1.97 |
| punopuno | Soft_N | 2.41 | 5.03 | 2.84 | 1.91 |
| bumyabumya | Soft_N | 2.28 | 5.38 | 3.16 | 2.31 |
| kadakada | Hard_N | 7.06 | 4.38 | 7.66 | 0.88 |
| gukuguku | Hard_N | 7.28 | 4.66 | 7.34 | 0.59 |
| godogodo | Hard_N | 7.91 | 5.66 | 7.28 | 1.97 |
| gokogoko | Hard_N | 7.13 | 4.75 | 7.34 | 0.75 |
| gukaguka | Hard_N | 6.97 | 4.78 | 7.09 | 1.31 |
| kagukagu | Hard_N | 6.78 | 4.81 | 6.81 | 0.97 |
| kakekake | Hard_N | 6.50 | 4.56 | 5.81 | 1.00 |
| gukoguko | Hard_N | 7.28 | 4.63 | 7.09 | 0.97 |
| gaigai | Hard_N | 7.19 | 4.22 | 7.22 | 0.38 |
| kogukogu | Hard_N | 6.72 | 4.19 | 5.97 | 1.28 |
| katokato | Hard_N | 6.13 | 3.84 | 5.47 | 0.97 |
| gotgot | Hard_N | 8.38 | 4.03 | 8.03 | 4.28 |
| tebahore | Pseudo | 5.00 | 4.94 | 5.50 | 0.06 |
| jizasaki | Pseudo | 5.97 | 4.81 | 7.47 | 0.09 |
| ruwagiku | Pseudo | 5.97 | 4.63 | 5.94 | 0.03 |
| wakosatu | Pseudo | 5.41 | 4.72 | 4.97 | 0.31 |
| kosochifu | Pseudo | 5.13 | 4.56 | 4.56 | 0.06 |
| machijiya | Pseudo | 5.53 | 5.06 | 5.41 | 0.03 |
| buranebo | Pseudo | 5.28 | 5.25 | 5.16 | 0.03 |
| gakigonu | Pseudo | 7.63 | 4.06 | 7.94 | 0.19 |
| iapeso | Pseudo | 4.41 | 4.38 | 4.09 | 0.06 |
| nibimuse | Pseudo | 4.88 | 4.72 | 5.53 | 0.06 |
| fugusau | Pseudo | 5.53 | 4.63 | 5.41 | 0.16 |
| shibaroto | Pseudo | 5.00 | 4.59 | 4.88 | 0.16 |

**Supplementary Table 3.** Mean Ratings for Each Word (Singaporean group)

| Words | Type | Hardness | Warmness | Roughness | Familiarity |
| --- | --- | --- | --- | --- | --- |
|  |  |  |  |  |  |
| funwaka | Soft_C | 4.64 | 4.95 | 4.68 | 1.27 |
| poyapoya | Soft_C | 2.73 | 5.41 | 3.23 | 1.18 |
| powapowa | Soft_C | 4.59 | 6.09 | 4.50 | 0.86 |
| fuwari | Soft_C | 3.00 | 6.14 | 3.50 | 2.23 |
| hoyahoya | Soft_C | 4.45 | 6.18 | 4.82 | 1.00 |
| fukafuka | Soft_C | 5.23 | 5.86 | 5.05 | 1.27 |
| pafupafu | Soft_C | 2.00 | 6.55 | 3.14 | 1.45 |
| funyafunya | Soft_C | 3.27 | 5.95 | 3.82 | 0.86 |
| puyopuyo | Soft_C | 3.32 | 5.50 | 3.14 | 0.55 |
| fuwafuwa | Soft_C | 2.95 | 6.32 | 2.91 | 2.23 |
| yuruyuru | Soft_C | 2.59 | 5.41 | 3.50 | 0.73 |
| kunyakunya | Soft_C | 4.50 | 5.18 | 4.77 | 0.91 |
| gachigachi | Hard_C | 6.05 | 5.86 | 5.14 | 1.41 |
| gacchingacchin | Hard_C | 6.91 | 4.95 | 5.86 | 0.86 |
| kachinkachin | Hard_C | 6.82 | 6.05 | 5.59 | 1.32 |
| gachingachin | Hard_C | 7.45 | 4.86 | 5.64 | 1.09 |
| kochikochi | Hard_C | 5.00 | 4.91 | 4.77 | 1.18 |
| gorigori | Hard_C | 4.86 | 5.05 | 4.32 | 1.23 |
| gichigichi | Hard_C | 4.59 | 4.91 | 6.00 | 0.50 |
| bakibaki | Hard_C | 5.82 | 5.55 | 5.32 | 0.73 |
| kachinkochin | Hard_C | 6.82 | 4.77 | 6.09 | 0.95 |
| kachikachi | Hard_C | 6.18 | 5.36 | 5.73 | 0.77 |
| kochinkochin | Hard_C | 5.91 | 4.73 | 6.05 | 0.68 |
| garigari | Hard_C | 4.59 | 5.45 | 4.91 | 0.73 |
| munumunu | Soft_N | 2.73 | 5.55 | 3.14 | 0.55 |
| munamuna | Soft_N | 2.86 | 5.45 | 2.91 | 0.68 |
| yapuyapu | Soft_N | 3.77 | 6.64 | 3.95 | 1.18 |
| payupayu | Soft_N | 3.95 | 6.09 | 4.00 | 1.18 |
| myunomyuno | Soft_N | 3.55 | 5.64 | 4.05 | 0.86 |
| fuyufuyu | Soft_N | 3.14 | 5.86 | 3.36 | 1.27 |
| fubafuba | Soft_N | 3.91 | 6.50 | 4.05 | 1.36 |
| funofuno | Soft_N | 3.05 | 5.59 | 3.32 | 0.45 |
| pohapoha | Soft_N | 3.32 | 6.09 | 3.95 | 0.55 |
| myofumyofu | Soft_N | 3.00 | 5.32 | 3.68 | 0.68 |
| punopuno | Soft_N | 3.18 | 4.82 | 3.18 | 0.64 |
| bumyabumya | Soft_N | 4.36 | 5.64 | 3.82 | 0.91 |
| kadakada | Hard_N | 6.55 | 5.09 | 6.05 | 1.45 |
| gukuguku | Hard_N | 5.23 | 5.05 | 4.64 | 1.41 |
| godogodo | Hard_N | 4.77 | 5.32 | 4.41 | 0.86 |
| gokogoko | Hard_N | 5.64 | 5.00 | 5.77 | 0.77 |
| gukaguka | Hard_N | 6.32 | 5.64 | 5.95 | 0.68 |
| kagukagu | Hard_N | 5.68 | 5.41 | 5.64 | 0.86 |
| kakekake | Hard_N | 6.50 | 5.18 | 5.64 | 1.09 |
| gukoguko | Hard_N | 5.23 | 4.91 | 5.32 | 0.86 |
| gaigai | Hard_N | 5.32 | 5.95 | 5.00 | 2.41 |
| kogukogu | Hard_N | 5.50 | 5.00 | 4.91 | 0.77 |
| katokato | Hard_N | 5.91 | 5.32 | 6.14 | 1.05 |
| gotgot | Hard_N | 5.91 | 5.18 | 5.64 | 0.68 |
| tebahore | Pseudo | 6.68 | 5.45 | 6.09 | 1.00 |
| jizasaki | Pseudo | 6.95 | 4.27 | 6.50 | 1.05 |
| ruwagiku | Pseudo | 5.95 | 4.59 | 5.82 | 1.00 |
| wakosatu | Pseudo | 5.36 | 5.23 | 6.36 | 1.23 |
| kosochifu | Pseudo | 3.64 | 4.91 | 3.55 | 1.05 |
| machijiya | Pseudo | 5.09 | 5.00 | 4.86 | 0.86 |
| buranebo | Pseudo | 4.64 | 4.91 | 4.91 | 0.86 |
| gakigonu | Pseudo | 6.50 | 4.86 | 5.50 | 1.45 |
| iapeso | Pseudo | 5.73 | 5.14 | 4.68 | 0.41 |
| nibimuse | Pseudo | 4.45 | 4.86 | 4.55 | 0.91 |
| fugusau | Pseudo | 5.41 | 5.00 | 4.41 | 0.73 |
| shibaroto | Pseudo | 4.95 | 4.95 | 5.32 | 0.86 |

**Supplementary Table 4.** Mean Ratings for Each Word (US group)

| US | Type | Hardness | Warmness | Roughness | Familiarity |
| --- | --- | --- | --- | --- | --- |
|  |  |  |  |  |  |
| funwaka | Soft_C | 4.48 | 4.74 | 4.52 | 0.61 |
| poyapoya | Soft_C | 3.04 | 6.57 | 2.43 | 0.61 |
| powapowa | Soft_C | 3.83 | 5.35 | 3.30 | 0.74 |
| fuwari | Soft_C | 3.09 | 6.04 | 2.43 | 1.39 |
| hoyahoya | Soft_C | 2.91 | 5.22 | 2.65 | 0.74 |
| fukafuka | Soft_C | 5.17 | 4.48 | 5.13 | 0.61 |
| pafupafu | Soft_C | 3.26 | 5.35 | 3.43 | 0.61 |
| funyafunya | Soft_C | 3.30 | 5.96 | 3.61 | 0.65 |
| puyopuyo | Soft_C | 2.17 | 5.87 | 2.83 | 1.61 |
| fuwafuwa | Soft_C | 2.39 | 6.39 | 2.96 | 1.09 |
| yuruyuru | Soft_C | 2.70 | 5.43 | 2.17 | 0.52 |
| kunyakunya | Soft_C | 6.00 | 5.17 | 5.17 | 0.04 |
| gachigachi | Hard_C | 4.39 | 5.70 | 4.22 | 0.65 |
| gacchingacchin | Hard_C | 6.09 | 4.09 | 6.43 | 0.57 |
| kachinkachin | Hard_C | 5.87 | 4.13 | 5.87 | 0.35 |
| gachingachin | Hard_C | 4.87 | 4.70 | 5.13 | 0.39 |
| kochikochi | Hard_C | 5.22 | 4.83 | 4.57 | 0.57 |
| gorigori | Hard_C | 4.52 | 5.57 | 3.70 | 0.83 |
| gichigichi | Hard_C | 4.48 | 5.61 | 4.74 | 0.30 |
| bakibaki | Hard_C | 5.57 | 5.35 | 4.26 | 0.83 |
| kachinkochin | Hard_C | 6.65 | 3.70 | 6.35 | 0.43 |
| kachikachi | Hard_C | 6.30 | 4.00 | 5.78 | 0.87 |
| kochinkochin | Hard_C | 7.30 | 3.78 | 5.83 | 0.39 |
| garigari | Hard_C | 5.13 | 4.30 | 4.48 | 0.52 |
| munumunu | Soft_N | 3.39 | 5.87 | 2.52 | 0.65 |
| munamuna | Soft_N | 2.09 | 6.22 | 2.17 | 0.70 |
| yapuyapu | Soft_N | 3.52 | 6.22 | 3.35 | 0.35 |
| payupayu | Soft_N | 2.52 | 5.70 | 2.61 | 0.57 |
| myunomyuno | Soft_N | 2.91 | 4.78 | 3.48 | 0.74 |
| fuyufuyu | Soft_N | 3.04 | 5.65 | 2.48 | 0.70 |
| fubafuba | Soft_N | 3.26 | 5.78 | 3.91 | 0.83 |
| funofuno | Soft_N | 2.43 | 6.13 | 3.00 | 0.70 |
| pohapoha | Soft_N | 3.00 | 5.30 | 3.43 | 0.30 |
| myofumyofu | Soft_N | 4.65 | 4.83 | 4.78 | 0.61 |
| punopuno | Soft_N | 3.70 | 5.39 | 3.74 | 0.74 |
| bumyabumya | Soft_N | 4.00 | 6.17 | 4.48 | 0.43 |
| kadakada | Hard_N | 6.91 | 3.43 | 6.57 | 0.39 |
| gukuguku | Hard_N | 5.61 | 4.13 | 5.91 | 0.26 |
| godogodo | Hard_N | 4.26 | 5.26 | 3.57 | 0.74 |
| gokogoko | Hard_N | 6.26 | 3.83 | 6.30 | 0.43 |
| gukaguka | Hard_N | 6.09 | 3.96 | 6.17 | 0.87 |
| kagukagu | Hard_N | 6.57 | 4.22 | 6.87 | 0.43 |
| kakekake | Hard_N | 6.30 | 4.00 | 6.13 | 0.57 |
| gukoguko | Hard_N | 6.17 | 4.17 | 6.13 | 0.48 |
| gaigai | Hard_N | 3.91 | 5.00 | 4.48 | 0.35 |
| kogukogu | Hard_N | 6.00 | 4.48 | 6.43 | 0.13 |
| katokato | Hard_N | 5.43 | 4.43 | 5.39 | 0.83 |
| gotgot | Hard_N | 5.52 | 4.52 | 4.35 | 0.70 |
| tebahore | Pseudo | 5.57 | 4.13 | 5.04 | 0.65 |
| jizasaki | Pseudo | 5.65 | 4.22 | 5.57 | 0.65 |
| ruwagiku | Pseudo | 6.13 | 3.43 | 6.26 | 0.13 |
| wakosatu | Pseudo | 5.74 | 5.09 | 5.65 | 0.35 |
| kosochifu | Pseudo | 4.74 | 4.52 | 5.43 | 0.26 |
| machijiya | Pseudo | 4.91 | 4.57 | 5.70 | 0.39 |
| buranebo | Pseudo | 4.87 | 4.78 | 4.26 | 0.57 |
| gakigonu | Pseudo | 5.91 | 3.78 | 5.78 | 0.43 |
| iapeso | Pseudo | 3.83 | 4.52 | 3.87 | 0.57 |
| nibimuse | Pseudo | 3.30 | 4.57 | 3.96 | 0.61 |
| fugusau | Pseudo | 4.09 | 4.78 | 4.00 | 0.39 |
| shibaroto | Pseudo | 5.35 | 4.52 | 4.74 | 0.09 |

**Supplementary Table 5.** Results of ANOVAs

| Effects | Japan (n = 32) | | |  | Singapore (n = 22) | | |  | United States (n = 23) | | |
| --- | --- | --- | --- | --- | --- | --- | --- | --- | --- | --- | --- |
|  | F | p | η^2^_p_ |  | F | p | η^2^_p_ |  | F | p | η^2^_p_ |
| Hardness rating | | | | | | | | | | | |
| Sound symbolism | 1054.85 | < 0.001 | 0.97 |  | 64.91 | < 0.001 | 0.76 |  | 45.4 | < 0.001 | 0.67 |
| Novelty | 16.82 | < 0.001 | 0.35 |  | 1.62 | 0.22 | 0.072 |  | 0.18 | 0.68 | 0.008 |
| Interaction | 251.4 | < 0.001 | 0.89 |  | 0 | 1 | 0 |  | 3.33 | 0.08 | 0.13 |
| Warmness rating | | | | | | | | | | | |
| Sound symbolism | 22.1 | < 0.001 | 0.42 |  | 0.006 | 0.94 | 0 |  | 19.55 | < 0.001 | 0.47 |
| Novelty | 17.95 | < 0.001 | 0.37 |  | 3.07 | 0.094 | 0.13 |  | 0.89 | 0.36 | 0.04 |
| Interaction | 44.06 | < 0.001 | 0.59 |  | 0.05 | 0.82 | 0.003 |  | 3.1 | 0.09 | 0.12 |
| Roughness rating | | | | | | | | | | | |
| Sound symbolism | 306.41 | < 0.001 | 0.91 |  | 29.57 | < 0.001 | 0.59 |  | 25.8 | < 0.001 | 0.54 |
| Novelty | 15.53 | < 0.001 | 0.33 |  | 2.95 | 0.10 | 0.12 |  | 5.59 | 0.027 | 0.2 |
| Interaction | 1.65 | 0.21 | 0.05 |  | 0.38 | 0.54 | 0.018 |  | 3.66 | 0.069 | 0.14 |
| Familiarity rating | | | | | | | | | | | |
| Sound symbolism | 0.638 | 0.43 | 0.02 |  | 0.058 | 0.81 | 0.003 |  | 1.64 | 0.21 | 0.069 |
| Novelty | 888.76 | < 0.001 | 0.97 |  | 3.40 | 0.08 | 0.14 |  | 1.46 | 0.24 | 0.062 |
| Interaction | 10.26 | 0.003 | 0.25 |  | 6.62 | 0.018 | 0.24 |  | 0.97 | 0.34 | 0.042 |
|  |  |  |  |  |  |  |  |  |  |  |  |

**Supplementary Table 6.** Regression Analysis Summary for All Three Cultures (with hardness ratings as dependent variable)

| Variable | Japanese | | | Singaporean | | | United States | | |
| --- | --- | --- | --- | --- | --- | --- | --- | --- | --- |
|  | β | t | P_FWE_ | β | t | P_FWE_ | β | t | P_FWE_ |
| Constant | 0.00^†^ | 0.00^†^ | 1 | 0.00^†^ | 0.00^†^ | 1 | 0.00^†^ | 0.00^†^ | 1 |
| /a/ | -0.03 | -0.15 | 1 | -0.17 | -0.65 | 1 | **-0.65** | **-2.73** | **0.032** |
| /b/ | 0.09 | 1.32 | 0.590 | 0.18 | 2.11 | 0.132 | 0.17 | 2.14 | 0.124 |
| /tʃ/ = ch | **0.36** | **3.17** | **0.011** | **0.42** | **3.09** | **0.013** | 0.30 | 2.41 | 0.068 |
| /d/ | 0.09 | 1.12 | 0.811 | 0.10 | 1.01 | 0.957 | **0.24** | **2.74** | **0.032** |
| /e/ | 0.02 | 0.23 | 1 | -0.07 | -0.75 | 1 | **-0.34** | **-3.65** | **0.003** |
| /f/ | -0.19 | -1.93 | 0.190 | -0.08 | -0.68 | 1 | **0.28** | **2.57** | **0.048** |
| **/g/** | **0.33** | **2.77** | **0.030** | **0.50** | **3.55** | **0.004** | **0.65** | **4.89** | **0.000*** |
| /h/ | -0.07 | -0.83 | 1 | 0.03 | 0.30 | 1 | 0.10 | 1.15 | 0.782 |
| /i/ | 0.05 | 0.24 | 1 | -0.46 | -1.92 | 0.195 | **-0.59** | **-2.65** | **0.039** |
| **/k/** | 0.12 | 0.98 | 1 | **0.50** | **3.33** | **0.007** | **1.18** | **8.41** | **0.000*** |
| /m/ | -0.01 | -0.08 | 1 | -0.05 | -0.46 | 1 | **0.26** | **2.70** | **0.035** |
| /n/ | -0.06 | -0.75 | 1 | 0.14 | 1.51 | 0.428 | 0.22 | 2.50 | 0.056 |
| /o/ | 0.00 | -0.01 | 1 | -0.40 | -1.43 | 0.491 | **-0.78** | **-2.95** | **0.019** |
| /p/ | -0.08 | -0.66 | 1 | -0.04 | -0.31 | 1 | **0.35** | **2.62** | **0.043** |
| /r/ | 0.03 | 0.37 | 1 | 0.00 | -0.02 | 1 | **0.22** | **2.58** | **0.046** |
| /t/ | 0.04 | 0.53 | 1 | 0.10 | 1.25 | 0.666 | 0.10 | 1.25 | 0.662 |
| /u/ | -0.14 | -0.58 | 1 | -0.60 | -2.12 | 0.128 | **-0.95** | **-3.59** | **0.004** |
| /w/ | -0.17 | -2.33 | 0.081 | 0.06 | 0.71 | 1 | 0.09 | 1.03 | 0.932 |
| /j/ = y | -0.20 | -2.34 | 0.080 | 0.12 | 1.16 | 0.769 | 0.22 | 2.23 | 0.102 |
| *p < 0.0005; † value < 0.005; P_FWE_ indicates p value Bonferroni corrected for multiple comparisons across three groups (p value times 3). Signs aligned for consistency across groups. | | | | | | | | | |
